# Unveiling the hidden role of SDHA in breast cancer proliferation: a novel therapeutic avenue

**DOI:** 10.1186/s12935-025-03746-6

**Published:** 2025-03-21

**Authors:** Liyun Yong, Yuan Fang, Lingli Jin, Xiuqin Zhang, Manuel A. Luis, Xiaoyan Lin, Shasha Tang, Fengfeng Cai

**Affiliations:** 1 Department of Breast Surgery, Tongji Hospital, School of Medicine, Tongji University, 389 Xincun Rd, Shanghai, 200065 China; 2 Department of Pathology, Tongji Hospital, School of Medicine, Tongji University, 389 Xincun Rd, Shanghai, 200065 China; 3 Department of Breast Surgery, Yangpu Hospital, School of Medicine, Tongji University, Shanghai, 200090 China

**Keywords:** Breast cancer, SDHA, Prognosis, Proliferation, Migration

## Abstract

**Background:**

We observed an increased presence of succinate dehydrogenase complex subunit A (SDHA), a mitochondrial enzyme, in breast cancer (BC), which contributes to its proliferation. While SDHA deficiency has been extensively researched in rare disorders, the upregulation of SDHA and its impact on BC remain understudied. The aim of this study is to investigate the role of SDHA in BC.

**Methods:**

The mRNA expression of SDHA was analyzed from TCGA, clinical BC tissues and various BC cell lines via qPCR. Immunohistochemistry was also applied to detect the SDHA expression. Our study investigated the functional outcomes of SDHA overexpression and knockdown in BC utilizing clinical BC tissues from patients and various BC cell lines (MDA-MB-453, MDA-MB-468, SKBR3, and MCF-7). Multiple web platforms and software tools, including R, HPA and TISIDB, were employed to perform comprehensive data analysis. SDHA overexpression and siSDHA were transiently transfected into the cancer cells separately to assess expression levels, cellular proliferation, and migration dynamics through colony formation assay, CCK8 assay, wound-healing analysis.

**Results:**

We found that the mRNA expression level of SDHA was higher in cancer tissues or cells than in non-cancerous tissues or mammary epithelial cell in TCGA dataset, BC clinical specimens and BC cell lines, respectively. High SDHA expression was associated with poor overall survival (OS, *p* = 0.016) and disease specific survival (DSS, *p* = 0.024) in BC patients. Besides, our findings revealed MDA-MB-468, SKBR3 and MCF-7 cells transfected with siSDHA exhibited significantly reduced proliferation and migration capabilities. Conversely, the proliferation and migration abilities of these BC cells significantly increased when transfected with SDHA overexpression.

**Conclusions:**

In conclusion, this study highlights the previously underestimated role of SDHA in BC proliferation, presenting a novel avenue for therapeutic intervention.

**Supplementary Information:**

The online version contains supplementary material available at 10.1186/s12935-025-03746-6.

## Introduction

Breast cancer (BC), a major health concern globally, remains one of the most common cancers affecting women and is the second leading cause of death due to cancer in women, as highlighted in the 2022 statistics by the American Cancer Society [1–3]. Presently, the clinical approach to managing BC predominantly hinges on well-established clinicopathological factors and molecular markers, such as the estrogen receptor, progesterone receptor, and human epidermal growth factor receptor 2 (HER2) [4, 5]. Accurate classification of BC into distinct molecular subtypes is crucial for effective clinical treatment [6]. However, the persistence of heterogeneity among tumor cells within a patient and across identical subtypes in different patients results in variations in prognosis and treatment responses [7–9]. The advent of precision medicine has intensified the demand for new prognostic biomarkers in BC. These biomarkers are crucial for categorizing patients with potentially unfavorable clinical outcomes and for tailoring individualized treatment strategies [10, 11]. Investigations into the prognosis of BC bear immense significance for affected patients. Recent advancements in molecular biology have revealed specific molecular biomarkers associated with the prognosis of BC patients, offering the potential for more accurate and effective prognostic assessments [12, 13].

Succinate dehydrogenase (SDH) is located in the inner mitochondrial membrane and is a crucial element of the mitochondrial respiratory chain. Among its subunits, SDH complex subunit A (SDHA) plays a vital role in cellular energy production [14, 15]. SDHA functions as a component of mitochondrial succinate coenzyme Q reductase (complex II) and actively participates in the tricarboxylic acid cycle and oxidative phosphorylation [16, 17]. Studies propose that dysregulation of SDHs may contribute to various diseases, including cancers, which have been frequently associated with paraganglioma, pheochromocytoma, gastrointestinal stromal tumors, and renal cell carcinoma [18, 19]. The loss of SDHA expression has been documented to be implicated in the occurrence of certain tumors [20–22]. Hence, it can be inferred that detecting SDHA gene expression may have diagnostic and prognostic value in certain cancers. Nevertheless, the relationship between SDHA and the prognosis of BC remains unclear.

In this investigation, we explored the expression levels of SDHA and its prognostic relevance in BC patients, drawing on data from The Cancer Genome Atlas (TCGA) and other accessible databases. We also conducted a range of experiments to validate the presence of SDHA in BC tissues and cell lines. Moreover, we manipulated BC cell lines to either suppress or amplify SDHA expression, aiming to elucidate its in vitro biological role. Our research points to a significant link between SDHA activity and the proliferation and migration of BC cells, suggesting its potential as a novel prognostic marker and a promising target for therapeutic intervention in BC.

## Materials and methods

### Bioinformatic dataset acquisition

The summary of SDHA protein expression in humans was retrieved from the Human Protein Atlas (HPA) at https://www.proteinatlas.org/. The Cancer Genome Atlas (TCGA) available at https://cancergenome.nih.gov was utilized for the analysis of SDHA mRNA expression in tumor samples dated prior to 2023, as well as their corresponding paracancer and normal samples [23]. The dataset comprising 33 cancers, including Adrenocortical carcinoma, Bladder Urothelial Carcinoma, Breast invasive carcinoma, Cervical squamous cell carcinoma and endocervical adenocarcinoma, Cholangiocarcinoma, Colon adenocarcinoma, Lymphoid Neoplasm Diffuse Large B-cell Lymphoma, Esophageal carcinoma, Glioblastoma multiforme, Head and Neck squamous cell carcinoma, Kidney Chromophobe, Kidney renal clear cell carcinoma, Kidney renal papillary cell carcinoma, Acute Myeloid Leukemia, Brain Lower Grade Glioma, Liver hepatocellular carcinoma, Lung adenocarcinoma, Lung squamous cell carcinoma, Mesothelioma, Ovarian serous cystadenocarcinoma, Pancreatic adenocarcinoma, Pheochromocytoma and Paraganglioma, Prostate adenocarcinoma, Rectum adenocarcinoma, Sarcoma, Skin Cutaneous Melanoma, Stomach adenocarcinoma, Testicular Germ Cell Tumors, Thyroid carcinoma, Thymoma, Uterine Corpus Endometrial Carcinoma, Uterine Carcinosarcoma, and Uveal Melanoma.

The RNA-sequencing data, originally formatted as Fragments Per Kilobase per Million, was converted and normalized to transcripts per million reads utilizing the Toil process. Subsequently, the data underwent log2 transformation to facilitate further analysis. Additionally, the evaluation of SDHA expression across different molecular subtypes was performed utilizing the “subtype” module within the TISIDB database [24].

## Correlation analysis between SDHA expression and clinicopathological characteristics of BC patients

To investigate the impact of the SDHA gene and clinical characteristics (such as age, sex, and stage) on BC prognosis, univariate and multivariate Cox regression analyses were implemented. The p-value, hazard ratio, and 95% confidence interval of each variable were displayed using the “forestplot” R package. The R software packages “Survival” and “Survminer” were utilized to perform Kaplan-Meier (KM) survival curve analysis. Kaplan-Meier plots, including overall survival (OS) and disease-specific survival (DSS), were generated utilizing the Kaplan-Meier Plotter (http://kmplot.com). The log-rank and Mantel-Cox tests were employed to compare high- and low-SDHA gene expression groups in BC [25].

## Bioinformatics analysis of SDHA mRNA expression in BC tissue

The 1222 BC samples were classified into two groups, namely high-SDHA expression and low-SDHA expression, based on the median value of SDHA expression. To analyze the differentially expressed genes (DEGs) between these groups, we employed the “DESeq2” (v1.26.0) R package, setting the log-fold change absolute value > 1.5 and the p-value < 0.05 as the threshold parameters. Furthermore, the “ggplot2” (v3.3.3) R package was utilized to generate heat maps depicting the DEGs.

## Functional enrichment analysis of SDHA

Gene Ontology (GO) function and Kyoto Encyclopedia of Genes and Genomes (KEGG) enrichment analyses were performed on genes closely interacting with SDHA. These genes were acquired from STRING, utilizing the “clusterProfiler” and “org.Hs.eg.db” packages in R. To determine the significance of the enrichment, we applied a p-value threshold of < 0.01 for both analyses. A bar chart was constructed utilizing the “ggplot2” package [26].

## Patients and samples

From March to October 2023, 20 BC tissues, along with their corresponding non-cancerous tissues, were harvested from patients who underwent surgery at Tongji Hospital, School of Medicine, Tongji University. Prior to surgery, none of the patients had received any neoadjuvant therapy, chemotherapy, or radiotherapy. The study received ethical approval from the Ethics Committee of Tongji Hospital and was conducted following the principles outlined in the Declaration of Helsinki. Prior to enrollment, all participants provided informed consent. After resection, all tissue samples were promptly preserved in liquid nitrogen.

### Cell culture

The BC cell lines MDA-MB-468, SKBR3, MCF-7, and MDA-MB-453, and the mammary epithelial cell line MCF10A were obtained from the Cell Bank of the Chinese Academy of Sciences. SKBR3, MDA-MB-468, MCF-7, and MDA-MB-453 cells were cultured in Dulbecco’s modified Eagle’s medium (Invitrogen), supplemented with 10% heat-inactivated fetal bovine serum (HyClone), 1% L-glutamine, 50 U/mL penicillin, and 50 mg/mL streptomycin. MCF10A cells were grown in MEGM Bullet Kit Growth Media supplemented with 100 ng/mL cholera toxin. All cell lines were maintained at 37 °C in a 5% CO2 environment and passaged utilizing standard cell culture techniques.

## Total RNA extraction and quantitative real-time PCR (qRT-PCR)

Total RNA isolation was completed using the RNAiso Plus Kit (Takara, catalog no. 9109). Subsequently, reverse transcription of RNA into cDNA was performed employing the PrimeScript RT Reagent kit (RR047, Takara). qPCR reactions were completed on a real-time PCR instrument (Roche, LightCycler 96, Switzerland) with a 20 µl reaction volume containing SYBR Premix Ex Taq (RR420, Takara) for expression determination, which was normalized to GAPDH. The qPCR primers, synthesized by Rui Mian Biological Technology (China), were as follows: SDHA-F: CTACAAGGGGCAGGTTCTGA and SDHA-R: TCTGCAATACTCAGGGCACA-3’. For GAPDH, the primers used were GAPDH-F: CATTGACCTCAACTACATGGTTT and GAPDH-R: GAAGATGGTGATGGGATTTCC. The 2-∆∆Ct method was utilized for mRNA expression quantification.

## Cell counting Kit-8 (CCK-8) analysis

Cell viability was evaluated by employing the CCK-8 kit (Beyotime, China). Briefly, MDA-MB-468, SKBR3, and MCF-7 cells were seeded in 96-well plates (2000 cells per well) and incubated overnight. At 24, 48, 72, and 96 h, 10 µl of CCK-8 solution was added to each well, followed by further incubation at 37 °C for 30, 60, 90, and 120 min. The absorbance at 450 nm was tested utilizing a Synergy H1 hybrid multimode microplate reader (BioTek Instruments Inc., USA) with Gen5 software (version 2.04).

### Wound-healing assay

The cells were seeded in 6-well plates (5 × 10^5^ cells per well) and cultured until reaching 90% confluence. To create wounds, the cell layers were gently scratched using a sterile 100 µL pipette tip. After that, the cells were rinsed twice with PBS to remove any detached cells and debris. Following the wash, the cells were cultured in a serum-free medium for 24 h. The closure of the wound was monitored and measured at specific time points.

### Colony formation assay

The transfected MDA-MB-468, SKBR3, and MCF-7 cells were initially seeded into 6-well plates (5 × 10^2^ cells per well). Subsequently, the cells were incubated at 37 °C in a 5% CO_2_ for 7–14 days, with the culture medium being refreshed every 3 days. Once colonies were formed, the cells were washed three times with phosphate-buffered saline before being fixed with 95% ethanol for 10 min. Following fixation, the colonies were stained with 0.1% crystal violet (Yeasen, China) for 10 min, and the number of colonies comprising ≥ 50 cells was counted under a microscope. Representative images were captured utilizing a camera.

### Lentivirus packaging and cell transfection

To achieve SDHA overexpression, SDHA was cloned into the lentiviral expression plasmid pLL3.7 (LTR-phCMV-EGFP-LTR). The lentivirus for SDHA overexpression was generated as follows: HEK-293T cells were seeded in a 15 cm culture dish (5 × 10^7^ cells ). After 16 h, PEI was chose to transfect the HEK-293T cells with plasmid DNA containing the target gene. The total amount of plasmid DNA used was 15 µg for the target plasmid pLY1 (LTR-phCMV-SDHA-HIS tag-EGFP-LTR) and two packaging plasmids, psPAX2 and pMD2G, in a ratio of 15 µg:15 µg:7.5 µg, respectively. The plasmid DNA was combined with 2 mL of serum-free culture medium and mixed thoroughly. Then, 112.5 µL of PEI (1 µg/mL), three times the total amount of plasmid DNA, was added, followed by mixation once again. After incubating at room temperature for 15 min, the mixture was added slowly to the HEK-293T cells. Following 60 h of transfection, the viral supernatant was collected by centrifugation at 2000 rpm for 10 min and filtered through a 0.45 μm filter membrane into sterilized virus centrifuge tubes. Subsequently, the virus was concentrated and purified by centrifugation at 4 °C and 15,000 rpm for 2 h.

Small interfering RNA (siRNA) with SDHA targeted siSDHA (siRNA-1: CAAGAGGGCATCTGCTAAA, siRNA-2: CCTCCAATTAAACCAAACG) or control siRNA for cell transfection were synthesized by RIBOBIO (Guangzhou, China). The transfection of MCF-7, SKBR3, and MDA-MB-468 cells with siRNA was carried out at a concentration of 100 nM employing Lipofectamine 3000 (Invitrogen).

### Western blot

The transfected BC cells were subjected to cell lysis using RIPA lysis buffer supplemented with 1% PMSF. Then, equal amounts of protein samples were subsequently separated on a 10% SDS-PAGE gel. Following separation, the protein bands were transferred from the gel to a PVDF membrane (Millipore, USA). Subsequently, the membranes were blocked and incubated with primary antibodies overnight at 4 °C, followed by incubation with secondary antibody for 1 h at room temperature. The primary antibodies used were GAPDH (GB15004-100, Servicebio, China), His tag (30402ES40, Yeasen, China), and the secondary antibody was Alexa Fluor labbled goat anti-Rabbit IgG (33119ES60, Yeasen, China).

### IHC

BC tissues were fixed utilizingg 4% paraformaldehyde and subsequently embedded in paraffin. The processed tissues were then sectioned into 4 μm slices and stained according to standard protocols. In this procedure, the primary antibody utilized was anti-SDHA (GB113278-100, Servicebio).

### Statistical analysis

Data analyses were completed utilizing GraphPad Prism (version 7.0). To evaluate expression differences between BC tissues and normal mammary epithelial tissues, the Mann-Whitney U test was utilized. The associations between SDHA expression and clinicopathologic characteristics of patients were analyzed utilizing either the Chi-square test or Fisher’s exact test. Survival curves were compared utilizing the log-rank test. For normally distributed data reported as mean ± SD, Student’s t-test was employed. Statistical significance was deemed as *p* < 0.05.

## Results

### Expression landscape and pan-cancer expression of SDHA

**Fig. 1:**
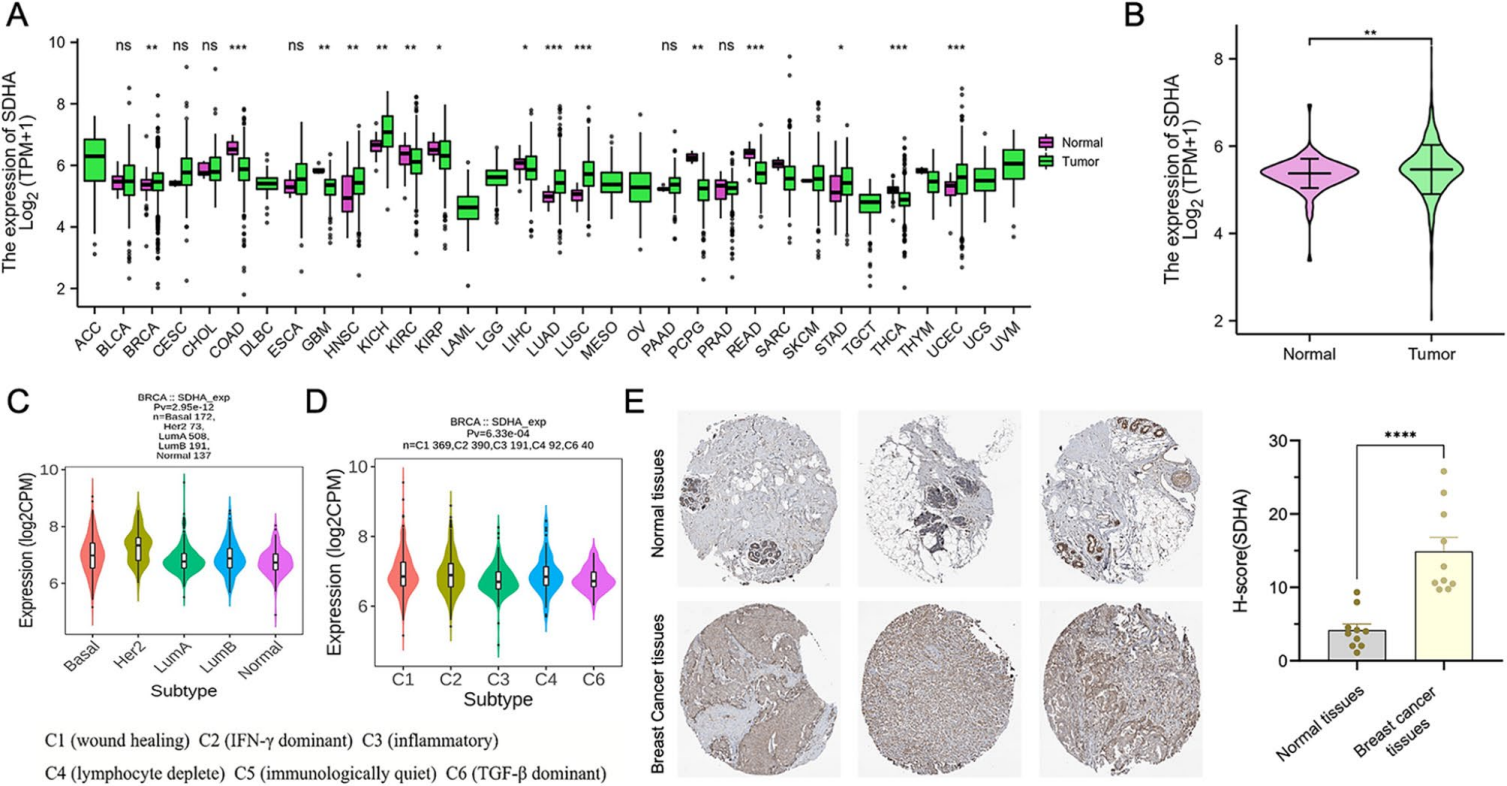
(**A**) TCGA database analysis showed the SDHA expression in 33 types of BC tissues and their corresponding adjacent normal tissues. ns, *p* ≥ 0.05; **p < 0.01; ***, *p* < 0.001. (**B**) Expression of SDHA in BC and normal breast tissues. (**C**) Correlations between SDHA expression and molecular subtypes. (**D**) Correlations between SDHA expression and immune subtypes. C1 (wound healing), C2 (IFN-γ dominant), C3 (inflammatory), C4 (lymphocyte deplete), C5 (immunologically quiet), and C6 (TGF-β dominant). (**E**) HPA database analysis showed representative IHC staining images of SDHA and quantitative bar chart in BC and non-cancerous tissues

SDHA mRNA expression was assessed across multiple cancer types. As depicted in Fig. 1A, high SDHA mRNA was noted in BRCA, HNSC, KICH, LUAD, LUSC, STAD, and UCEC (all *p* < 0.01) compared to normal samples. Analysis for ACC, DLBC, LAML, LGG, MESO, OV, TGCT, UCS, and UVM was not feasible due to a lack of sufficient normal samples. In BLCA, CESC, CHOL, ESCA, PAAD, and PRAD, no marked differences were observed (*p* > 0.05). Notably, SDHA mRNA expression was higher in BRCA compared to adjacent tissue (*p* < 0.01) (Fig. 1B). Additional examination of SDHA expression in both immune and molecular BC subtypes was performed, demonstrating a rise in SDHA expression, particularly in HER2-positive BC subtypes (Fig. 1C), and notable variations across the five immune subtypes (Fig. 1D). Protein-level data for SDHA, sourced from the HPA database, indicated a higher presence of SDHA protein in BC tissues compared to normal counterparts (Fig. 1E).

### SDHA is expressed at upregulated levels in BC

**Fig. 2:**
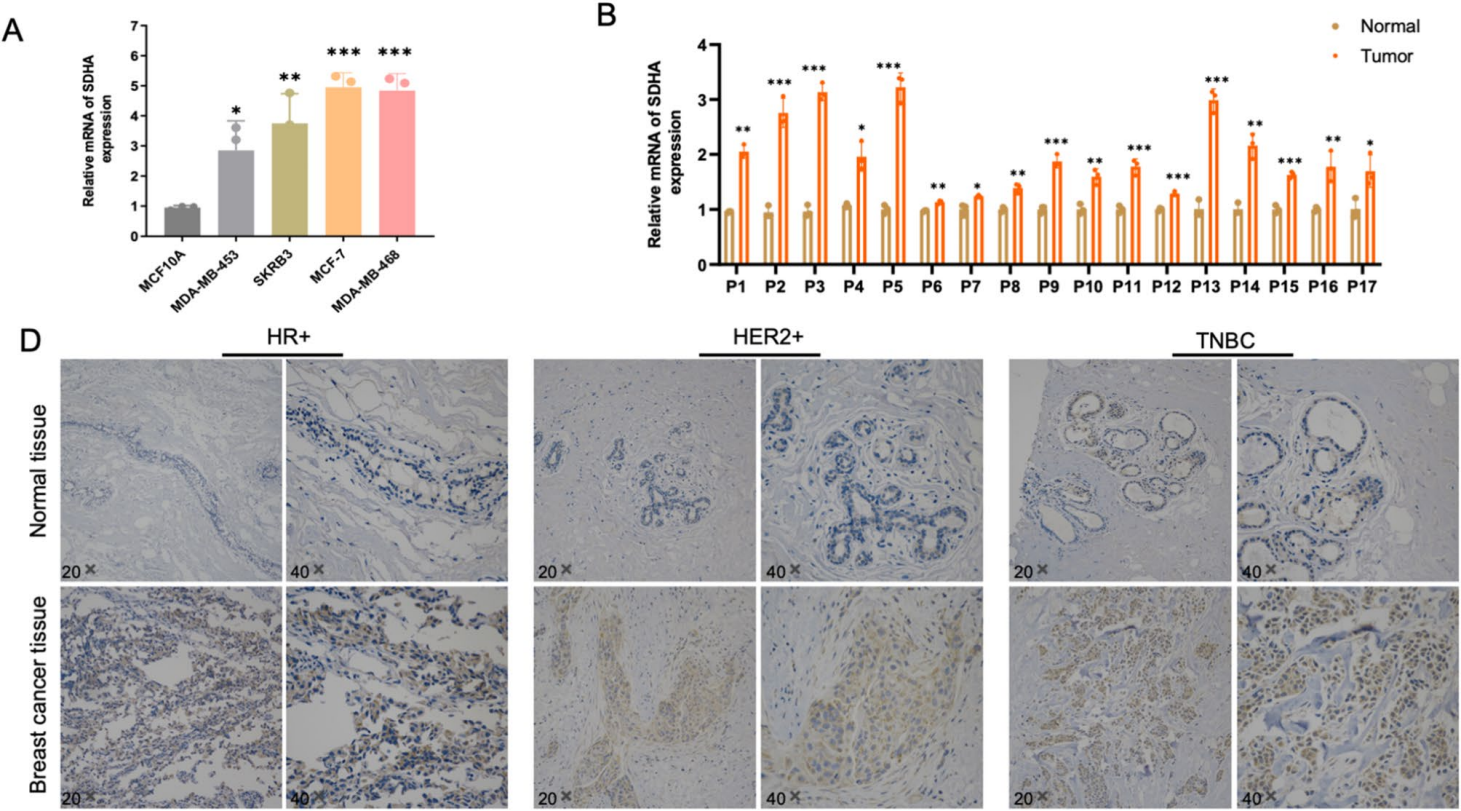
The expression of SDHA in BC was verified in vitro. (**A**) Quantitative RT-PCR analysis of SDHA mRNA expression in different BC cell lines and MCF10A cells. (**B**) Quantitative RT-PCR analysis of SDHA mRNA expression in 20 paired BC and non-cancerous tissues. ns, *p* ≥ 0.05; **p < 0.01; ***, *p* < 0.001; ****, *p* < 0.0001. (**C**) Representative IHC staining image of SDHA in different molecular subtypes of pared BC tissues and non-cancerous tissues

To validate the expression of SDHA in BC, a series of experiments were conducted on patient tissues and various cell lines. Concurrent with the previous findings, qPCR determination demonstrated a significantly higher expression of SDHA in the enrolled BC cells than MCF-10 A cells (Fig. 2A). Additionally, Fig. 2B illustrated higher expression of SDHA in the majority of clinical BC tissues (17 out of 20 matched tumor/normal tissues analyzed). Immunohistochemistry (IHC) further confirmed the upregulation of SDHA in HR + BC, HER2 + BC and TNBC patients (Fig. 2C). Collectively, these findings demonstrate that SDHA expression is markedly elevated in BC tissues.

### Evaluation of prognostic relevance of SDHA in BC

**Fig. 3:**
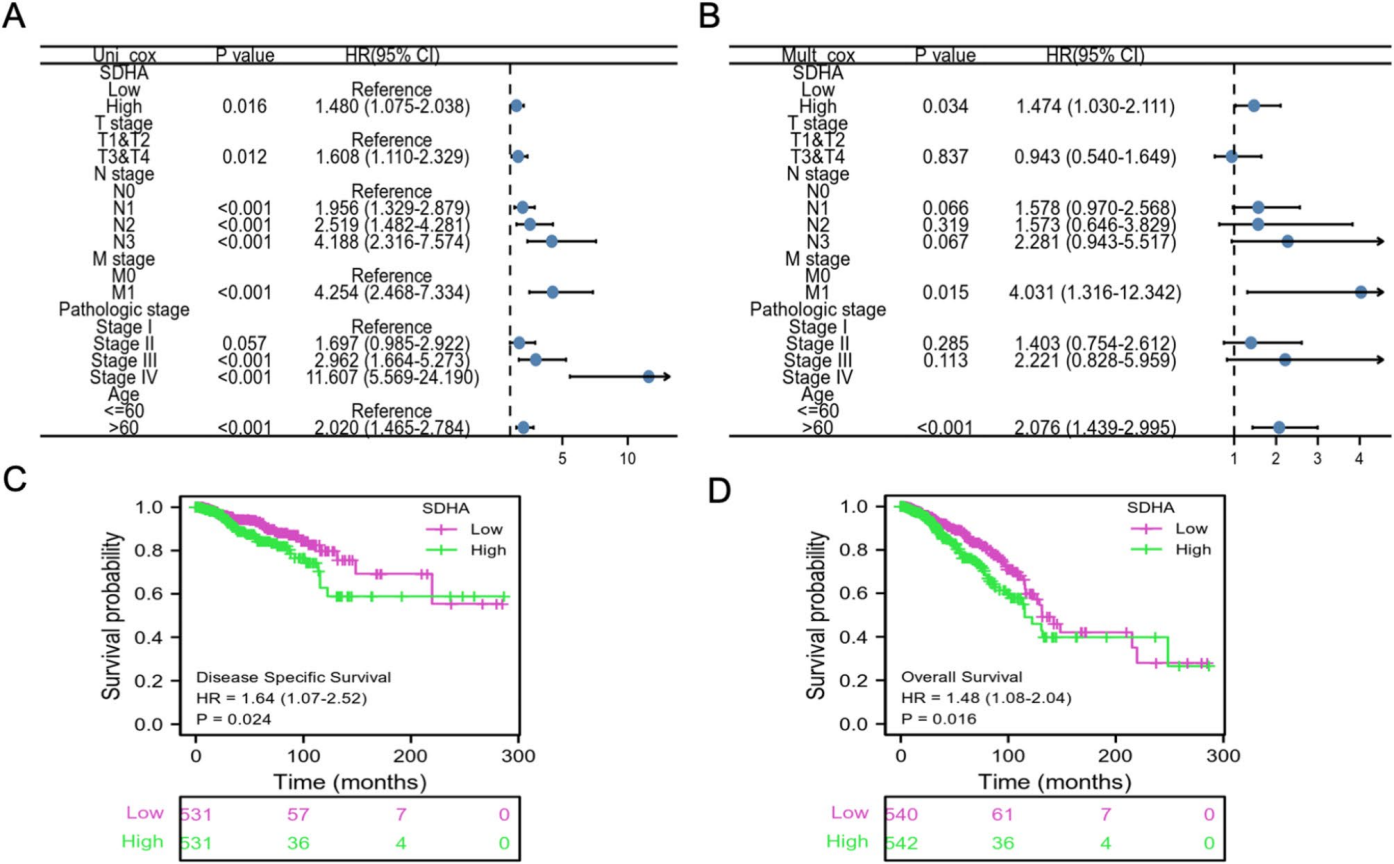
SDHA showed a poor prognostic prediction value in BC patients. A-B Univariate (**A**) and multivariate (**B**) Cox regression analysis results showed that SDHA was a significant prognosis risk factor of BC. **C**-**D** The Kaplan-Meier plotter database analysis shows the differences in (**C**) disease-specific survival and (**D**) overall survival of BC patients with high- and low-SDHA expression

Initially, a forest plot integrating univariate and multivariate Cox regression analyses was employed to examine the correlation between SDHA expression, clinical factors (such as age, pTNM stage, and pathologic stage), and OS in BC patients. Univariate Cox analysis revealed significant associations between high SDHA expression (*p* = 0.016), pN3 stage (*p* < 0.001), pM1 stage (*p* < 0.001), and pathologic stage III & IV with OS in BC. Furthermore, in the multivariate Cox regression analysis, SDHA emerged as an independent prognostic factor for BC (*p* = 0.034) (Fig. 3A, B). Building upon these findings, we further explored the relationship between SDHA expression and survival outcomes in the BRCA cohort, depicted through Kaplan-Meier survival curves. The KM survival analysis demonstrated that BC patients with high SDHA expression revealed lower OS (*p* = 0.016) and DSS (*p* = 0.024) than those with low SDHA expression (Fig. 3C, D). Collectively, these results highlight the association of elevated SDHA expression with poor prognosis in BC patients, suggesting that SDHA may serve as a potential prognostic marker for BC.

### Functional enrichment analysis of SDHA-associated DEGs in BC

**Fig. 4:**
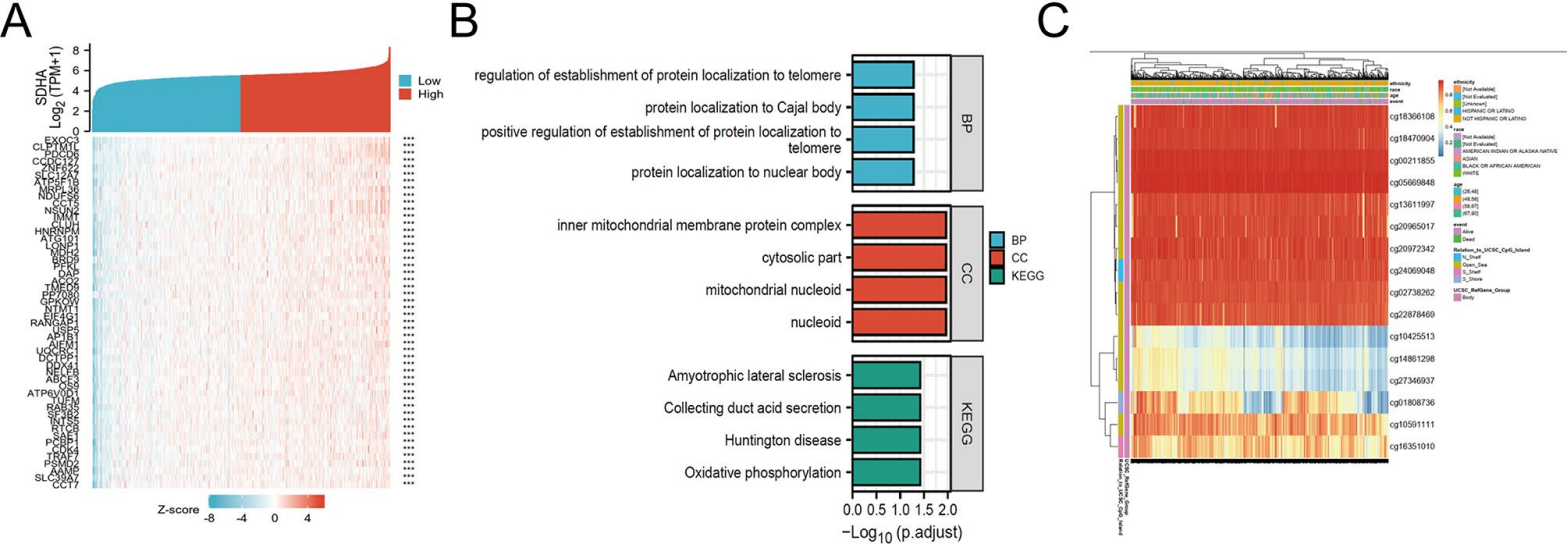
(**A**) The heat maps showed the expression of specific mRNAs in the BC patients with high- and low-SDHA expression from the TCGA-BC project. (**B**) GO/KEGG pathway enrichment for SDHA. GO/KEGG enrichment analyses were performed on these genes. (**C**) DNA methylation levels in the SDHA gene were associated with the prognosis of BC patients

To functionally annotate the DEGs associated with SDHA in BC patients, we utilized the “clusterProfiler” R package. The GO enrichment analysis results, which encompassed highly enriched biological processes, cellular components, and KEGG pathways, are depicted in Fig. 4A. Specifically, the top biological processes were “regulation of establishment of protein localization to telomere,” “protein localization to Cajal body,” “positive regulation of establishment of protein localization to telomere,” and “protein localization to nuclear body.” In terms of cellular components, significant enrichment was observed in the “inner mitochondrial membrane protein complex,” “cytosolic part,” “mitochondrial nucleoid,” and “nucleoid.” The top KEGG pathways included Amyotrophic lateral sclerosis, Collecting duct acid secretion, Huntington’s disease, and Oxidative phosphorylation. A heat map was generated to visualize the co-expression of SDHA with its mRNA counterparts (Fig. 4B). Notably, positive correlations were observed between SDHA expression and all the analyzed genes.

### Methylation status of the SDHA gene is linked to the prognosis of BC patients

The MethSurv tool was utilized to analyze DNA methylation levels in the SDHA gene and assess the prognostic value of CpG islands within the gene. The analysis revealed 16 methylated CpG islands, including cg18366180, cg18470904, cg00211855, cg05669848, cg13611997, cg20965017, cg20972343, cg24069048, cg02738262, and cg22878469 (Fig. 4C). Moreover, the methylation levels of three specific CpG islands, namely cg10425513, cg18366108, and cg24069048, were linked to prognosis (*p* < 0.05) (Table 1). Notably, increased methylation levels in these three CpG islands, particularly cg24069048, were linked to poor OS in BC patients than those with lower CpG methylation levels in the SDHA gene.

**Table 1:**
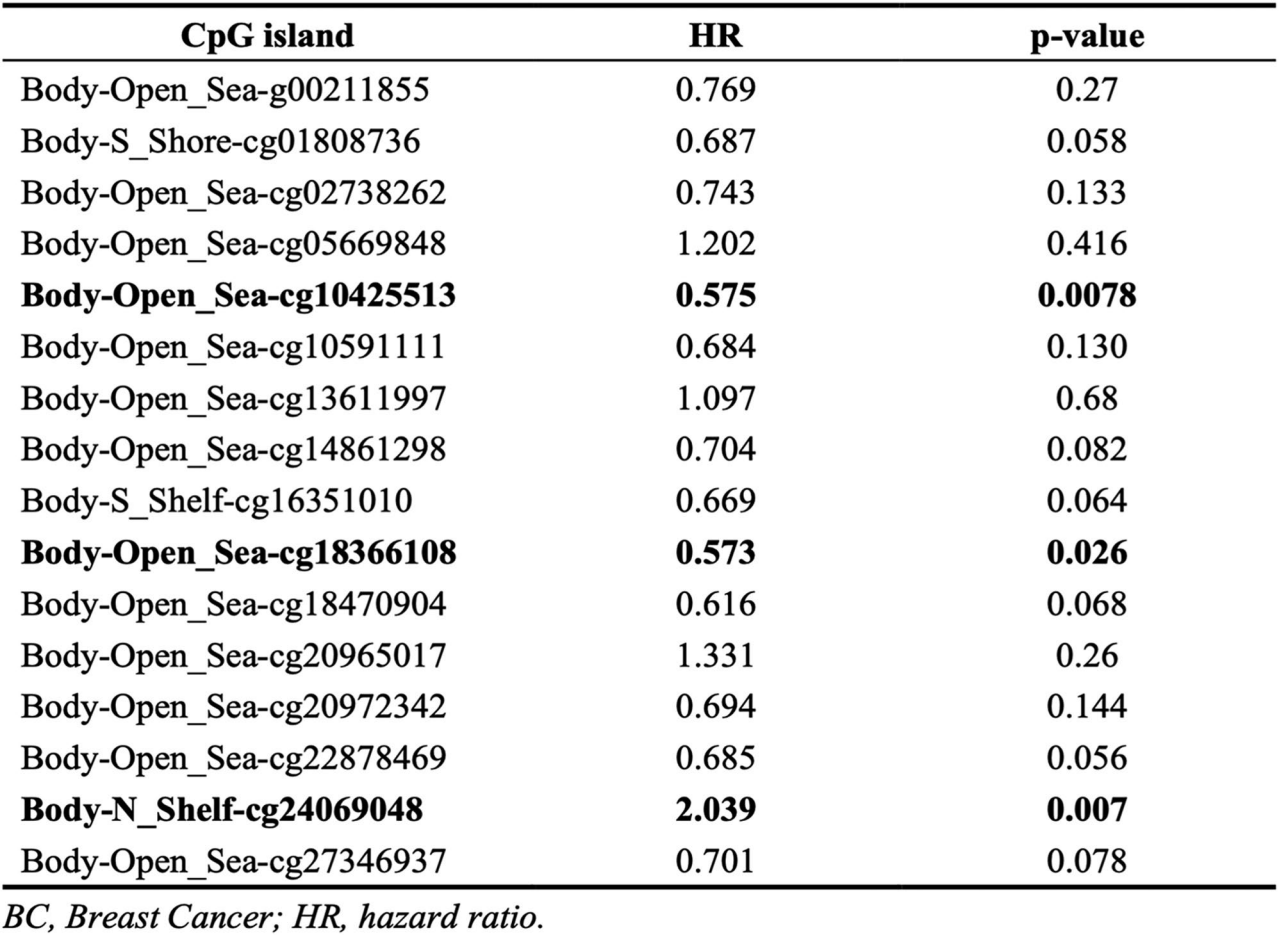
Effects of methylation levels in the CpG sites of the SDHA gene on the prognosis of BC patients

#### Knockdown of SDHA inhibits BC cell proliferation and migration in vitro

**Fig. 5:**
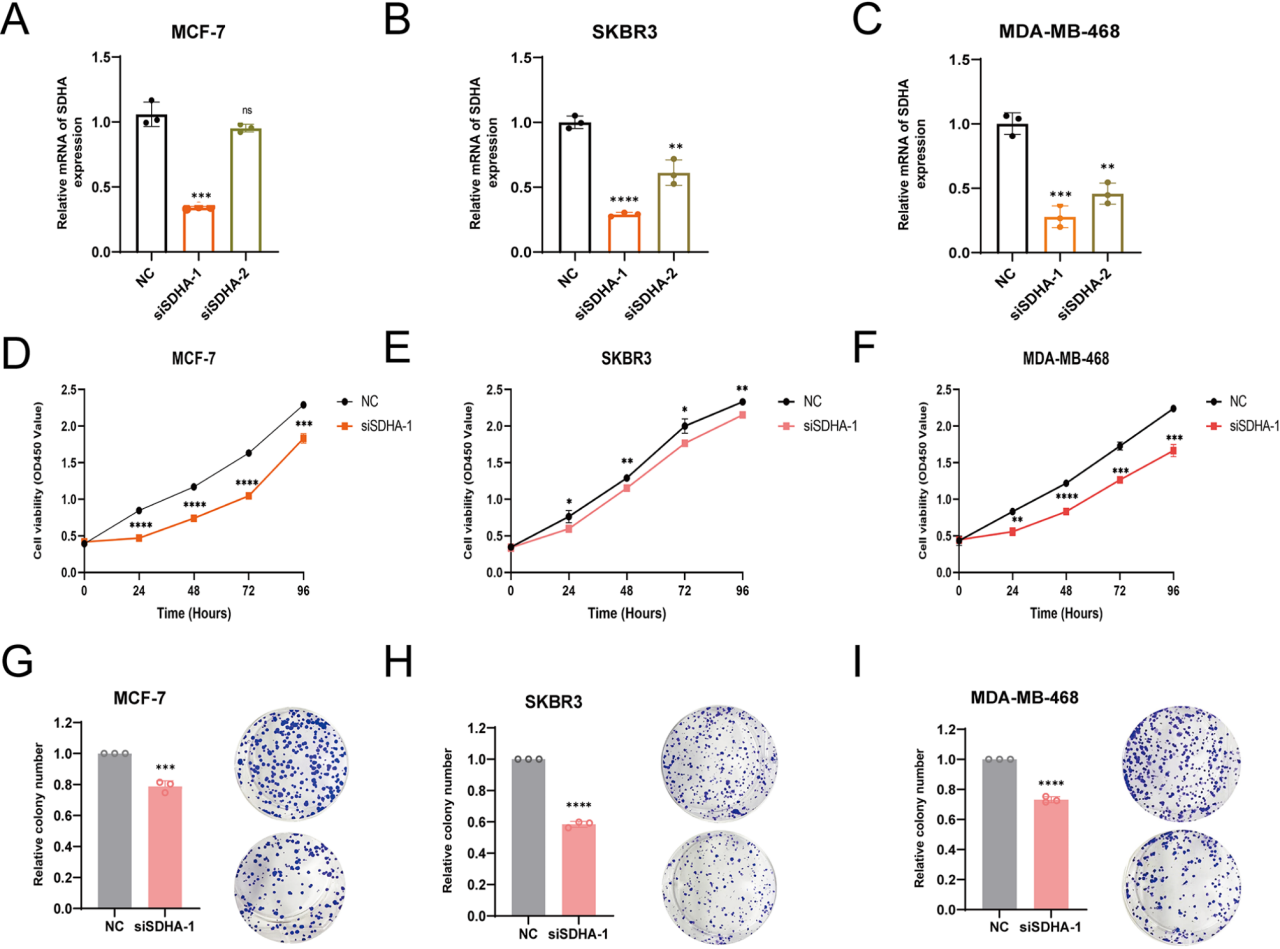
Abolishing expression of SDHA inhibited BC cell growth and migration. (**A**-**C**) The mRNA levels of SDHA in MCF-7, SKBR3 and MDA-MB-468 cells with SDHA knockout were measured by qPCR. (**D**-**F**) CCK-8 assays were performed to detect the proliferative capacity of MCF-7, SKBR3 and MDA-MB-468 cells with SDHA knockout. (**G**-**I**) Colony formation assays were conducted to determine the colony-forming abilities of MCF-7, SKBR3 and MDA-MB-468 cells with SDHA knockout

To assess the biological function of SDHA in BC cells, transfections of MCF-7, SKRB3, and MDA-MB-468 cells were conducted utilizing siRNA. To account for the potential off-target effects of siRNAs, we employed SDHA-siRNA1 and SDHA-siRNA2. The effectiveness of SDHA knockdown was measured using qPCR (Fig. 5A-C). Subsequently, evaluation of changes in cell functions indicated that the knockdown of SDHA led to a significant decline in the proliferation activity and colony formation ability of MCF-7, SKRB3, and MDA-MB-468 cells (Fig. 5D-I). However, due to growth conditions, we only performed the cell migration analysis on MCF-7 and MDA-MB-468 cells, excluding SKBR3 cells. Supporting this, the results obtained from the wound-healing assay demonstrated that loss of SDHA function decreased the migration ability of these two cell lines (Supplementary Fig. 1A, B). In conclusion, our findings suggest that loss-of-function of SDHA inhibits both the proliferation and migration abilities of BC cells.

### Restored SDHA promotes BC cell proliferation and migration

**Fig. 6:**
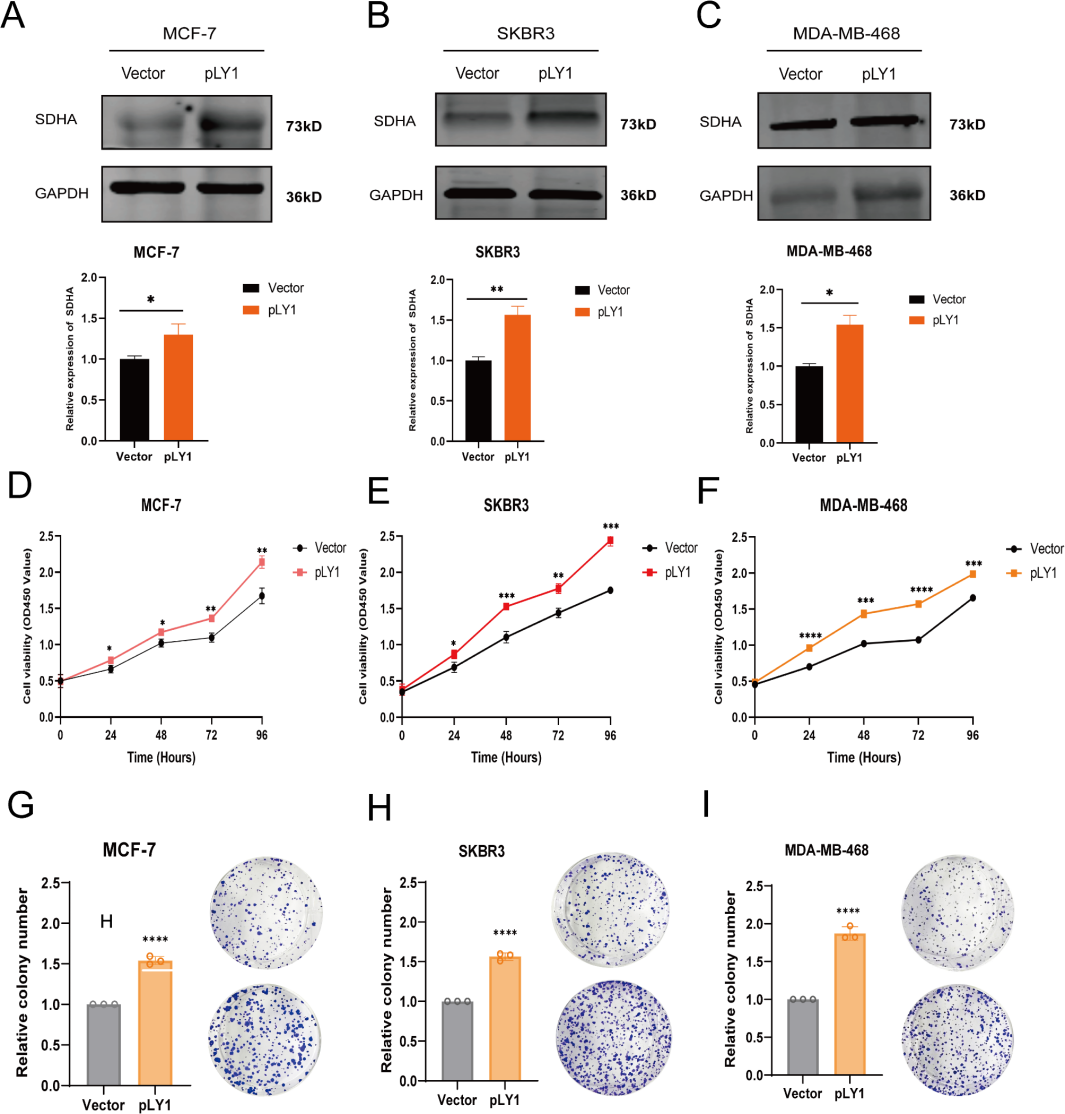
Restoring expression of SDHA promoted BC cell growth and migration in vitro. (**A**-**C**) The mRNA and protein levels of SDHA in MCF-7, SKBR3 and MDA-MB-468 cells with SDHA overexpression were measured by western blot and qRT-PCR. (**D**-**F**) CCK-8 assays were performed to compare the proliferative capacity of MCF-7, SKBR3 and MDA-MB-468 cells after overexpression of SDHA. (**G**-**I**) Colony formation assays were conducted to compare the colony-forming abilities of MCF-7, SKBR3 and MDA-MB-468 cells after overexpression of SDHA. *p < 0.05, **p < 0.01, ***p < 0.001, ****p < 0.0001

To ascertain the biological function of SDHA in BC cells, we generated stable BC cell lines expressing SDHA (Supplementary Fig. 2A, B, C). Firstly, we confirmed SDHA expression in the stably transfected cells through western blot analysis (Fig. 6A-C). Subsequently, a series of in vitro experiments were conducted to investigate the role of SDHA in BC cells. The CCK8 assay revealed a significant elevation in cell proliferation upon SDHA overexpression in the MCF-7, SKRB3, and MDA-MB-468 cell lines (Fig. 6D-F). Additionally, the long-term effects of SDHA on BC cell proliferation were assessed using colony formation experiments, where the SDHA overexpression led to a higher number of colonies (Fig. 6G-I). Furthermore, the impact of SDHA was evaluated on BC cell migration and invasion, specifically in MCF-7 and MDA-MB-468 cells. Notably, SDHA overexpression greatly enhanced the migration abilities of MCF-7 and MDA-MB-468 cells (Supplemental Fig. 3A, B). Collectively, these in vitro findings establish SDHA as a critical oncogene that promotes tumorigenesis in BC.

Furthermore, the mRNA expression of relevant genes was analyzed in MCF-7, SKBR3, and MDA-MB-468 cells following the overexpression of SDHA using qPCR. The genes studied included SNAIL1, SNAIL2, CDH1, CDH2, CDK1, CCNB, VIM, Twist1, Caspase3, Caspase9, Bax, Bcl2, Ndufs1, PGC1, Esrra, and CTGF. Our findings revealed simultaneous upregulation of SNAIL1, SNAIL2, CDH1, Caspase9, and CTGF genes in MCF-7, SKBR3, and MDA-MB-468 cells upon SDHA overexpression, whereas CDK1 exhibited reduced expression (Supplemental Fig. 4A, B, C).

## Discussion

This study explored the implications of overexpression and knockout of SDHA in various BC cell lines, aiming to understand their impact on the tumor phenotype. Our findings revealed a significant upregulation of SDHA expression in BC tissues, as observed in the TCGA and a validated cohort of 20 paired BC tissues and diverse cell lines. Importantly, the overexpression of SDHA correlated with poor OS, indicating its potential as a novel prognostic biomarker for BC. Furthermore, our investigation demonstrated that the loss of SDHA function greatly inhibited the malignant biological characteristics of BC, including cell growth and migration. Conversely, the overexpression of SDHA substantially promoted cancer cell proliferation and metastasis. These results highlight SDHA as a promising therapeutic target for intervention in BC.

SDH plays a pivotal role in linking succinate oxidation to fumarate and the conversion of ubiquinone into ubiquinol. This linkage is key in integrating the mitochondrial electron transport chain with the tricarboxylic acid cycle [27]. Comprising four subunits, the SDH complex includes SDHA, functioning as the flavoprotein, and SDHB, which serves as the iron-sulfur protein component. These subunits form the catalytic core and attach intricately to the inner mitochondrial membrane via SDHC and SDHD proteins, creating the entire protein complex [28]. The operational integrity of the SDH complex depends on the precise integration of these elements [29]. Genetic alterations in any of the SDH subunits can disrupt this assembly, leading to a spectrum of clinical conditions, ranging from neurodevelopmental disorders to rare cancers [16, 27, 30, 31]. An overabundance of succinate can hinder HIF1α prolyl hydroxylase activity, resulting in HIF-1α stabilization and elevated transcription of HIF1α-regulated genes, thereby aiding in cancer development [27]. Moreover, an impaired assembly of complex II often causes an accumulation of unbound SDHA flavoprotein in the mitochondrial matrix [32].

Prior research has established a connection between the buildup of succinate and tumor genesis in cases of SDH deficiency. Additionally, studies have pinpointed SDHA as a potential tumor suppressor in various cancers, including hepatocellular cancer and paraganglioma [14, 16]. While SDH subunit mutations are relatively rare in BC, the amplification or heightened expression of the SDHA subunit is more common, observed in about 20% of such tumors. Nevertheless, the specific consequences of SDHA overexpression and its influence on BC are not yet fully understood, underscoring the necessity for more in-depth investigation.

A study has uncovered that elevated SDHA causes metabolic enhancement, marked by an increase in mitochondrial respiration and glycolysis, resulting in a higher ATP production rate, as seen in ovarian cancer [33]. While previous research has extensively investigated SDH deficiency in rare disorders and malignancies [16, 30], studies on SDHA gain-of-function are limited. Elevated SDHA activity is linked with enhanced mitochondrial respiration and the accumulation of fumarate, impacting the metabolic processes in certain diseases [34, 35]. For example, individuals with primary antibody deficiency syndrome have been observed to display an SDHA gain-of-function phenotype, leading to an inflammatory reprogramming of B lymphocytes. This change triggers systemic inflammation and intensifies the severity of the condition [34]. Similarly, in metastatic uveal melanoma, increased SDHA levels contribute to metabolic imbalances, signified by heightened mitochondrial respiration. Such metabolic shifts are correlated with resistance to therapy, subsequently reducing the time until metastasis and decreasing patient survival rates [35]. Consistent with previous findings [35], this study revealed a positive correlation between tumor grade and SDHA expression in the tumor stroma of phyllodes tumors in the breast. This observed trend is hypothesized to be linked to the heightened metabolic demand in high-grade tumors [36]. Furthermore, our data demonstrated that elevated SDHA expression showed linkage with a poorer prognosis in BC patients with a high malignant grade. This finding was supported by IHC analysis of BC tissues obtained from clinical patients, which yielded similar results. However, it is noteworthy that SDHA can also function as a tumor suppressor, as it was frequently found to be decreased in 56% of patients with hepatocellular carcinoma (HCC) tissues. This research provides initial direct evidence suggesting that the loss of SDH activity plays a tumor-promoting role in HCC models by inducing the accumulation of cellular succinate (SDHA/B in HCC) [14].

The role of epigenetic alterations in the development and progression of cancerous tumors is increasingly acknowledged, particularly DNA methylation in gene promoters, leading to gene expression suppression or silencing [37, 38]. Recent research has highlighted the connection between abnormal methylation of CpG islands and various aspects of BC, including gene expression loss, developmental pathways, chemotherapy response, and the control of cancer stem cells [39–41]. Identifying new tumor suppressor genes activated by promoter methylation could offer novel diagnostic and therapeutic evaluation strategies. In our study, an in-depth analysis of TCGA data revealed abnormal hypermethylation in the SDHA gene’s promoter CpG islands. Additionally, we discovered three specific CpG islands in SDHA exhibiting hypomethylation, which was associated with a more favorable prognosis in BC.

Unfortunately, this article does not provide a mechanistic study on the role of SDHA overexpression in promoting BC proliferation. However, according to the KEGG data, this process may be linked to oxidative phosphorylation. Previous studies have shown that invasive cancer cells utilize transcriptional coactivators like peroxisome proliferator-activated receptor gamma coactivator 1-alpha (PGC-1α) to enhance oxidative phosphorylation, mitochondrial biogenesis, and oxygen consumption rates [42]. Therefore, we propose that SDHA might interact with PGC-1α to facilitate BC occurrence and progression. Further investigation is needed to explore this aspect of research in the future. Additionally, the functional experiments in this study were primarily conducted in vitro using BC cell lines. While these models provide valuable preliminary data, they may not fully recapitulate the complexity of the tumor microenvironment (TME), which includes interactions with stromal cells, immune cells, and extracellular matrix components. Future studies should include in vivo models, such as xenograft or genetically engineered mouse models, to validate these findings and better understand the role of SDHA in the context of the TME.

In summary, our study provides new insights into the impact of SDHA upregulation on BC proliferation. It is important to consider the individual patient characteristics and the heterogeneity of BC when assessing the influence of SDHA upregulation on cancer progression. Our findings suggest that the metabolic phenotype associated with SDHA upregulation may contribute to increased proliferation rates in BC, potentially involving elevated energy production, altered metabolism, reactive oxygen species production, epigenetic regulation, and cell signaling pathways. Further research is needed to fully understand the specific molecular mechanisms and signaling pathways affected by SDHA in BC, which could ultimately inform the development of targeted therapeutic strategies. Moving forward, we will prioritize elucidating the mechanistic aspects that promote tumor proliferation in SDHA-elevated BC cells. This work represents a step forward in our long-term goal of exploring innovative strategies to selectively target the metabolism of BC cells and improve treatment options for BC patients.

## Electronic supplementary material

Below is the link to the electronic supplementary material.

Supplementary Material 1: Supplementary Fig. 1. The wound-healing assay indicated that the loss of function of SDHA reduced the migration ability of MDA-MB-468 (A) and MCF-7 cells (B).

Supplementary Material 2: Supplementary Fig. 2. The relative expression of SDHA in MCF-7 (A), SKBR3 (B) and MDA-MB-468 (C) breast cancer cells after stable transfer of SDHA overexpression vector.

Supplementary Material 3: Supplementary Fig. 3. Wound-healing assay indicated that overexpression of SDHA promoted the migration ability of MDA-MB-468 (A) and MCF-7 cells (B).

Supplementary Material 4: Supplementary Fig. 4. mRNA expression of related genes in these cells after overexpression of SDHA in MCF-7 (A), SKBR3 (B) and MDA-MB-468 (C) cells.

## Data Availability

No datasets were generated or analysed during the current study.
