# Supplementary figures and images for: Unveiling the hidden role of SDHA in breast cancer proliferation: a novel therapeutic avenue
Source: Cancer Cell Int. 2025 Mar 21;25:108. doi: 10.1186/s12935-025-03746-6 (PMC11927305; doi:10.1186/s12935-025-03746-6)

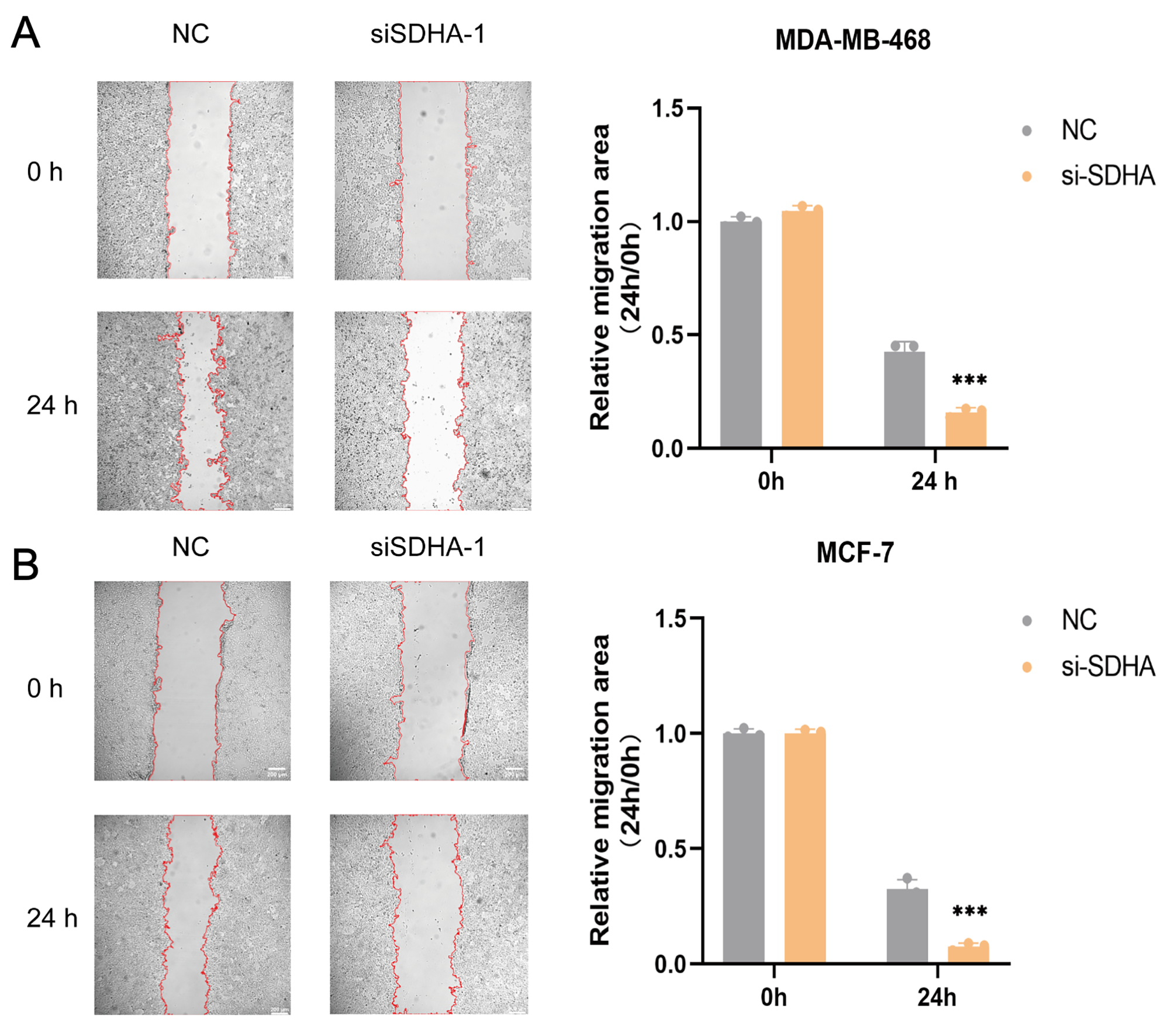

Supplement: Supplementary file 1 — Supplementary Material 1: Supplementary Fig. 1. The wound-healing assay indicated that the loss of function of SDHA reduced the migration ability of MDA-MB-468 (A) and MCF-7 cells (B). [file 12935_2025_3746_MOESM1_ESM.tiff]

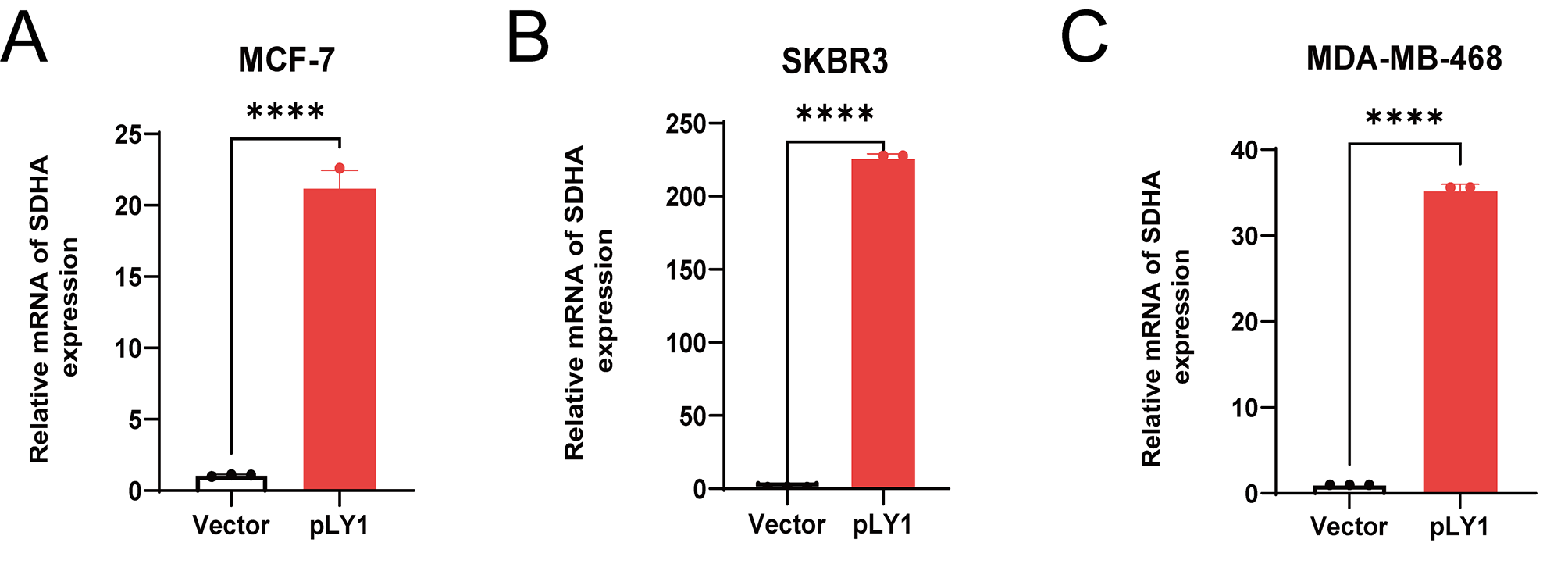

Supplement: Supplementary file 2 — Supplementary Material 2: Supplementary Fig. 2. The relative expression of SDHA in MCF-7 (A), SKBR3 (B) and MDA-MB-468 (C) breast cancer cells after stable transfer of SDHA overexpression vector. [file 12935_2025_3746_MOESM2_ESM.tiff]

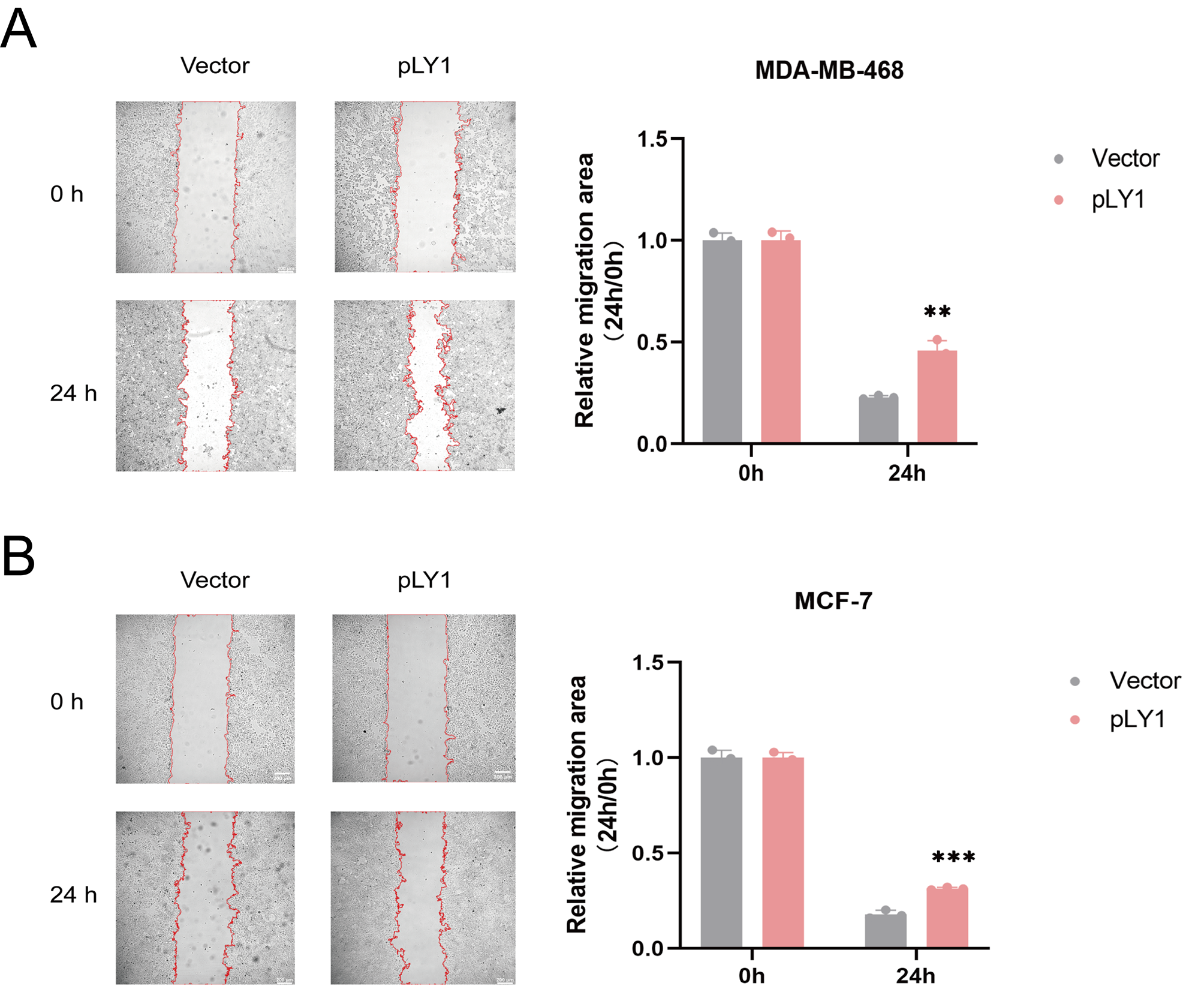

Supplement: Supplementary file 3 — Supplementary Material 3: Supplementary Fig. 3. Wound-healing assay indicated that overexpression of SDHA promoted the migration ability of MDA-MB-468 (A) and MCF-7 cells (B). [file 12935_2025_3746_MOESM3_ESM.tiff]

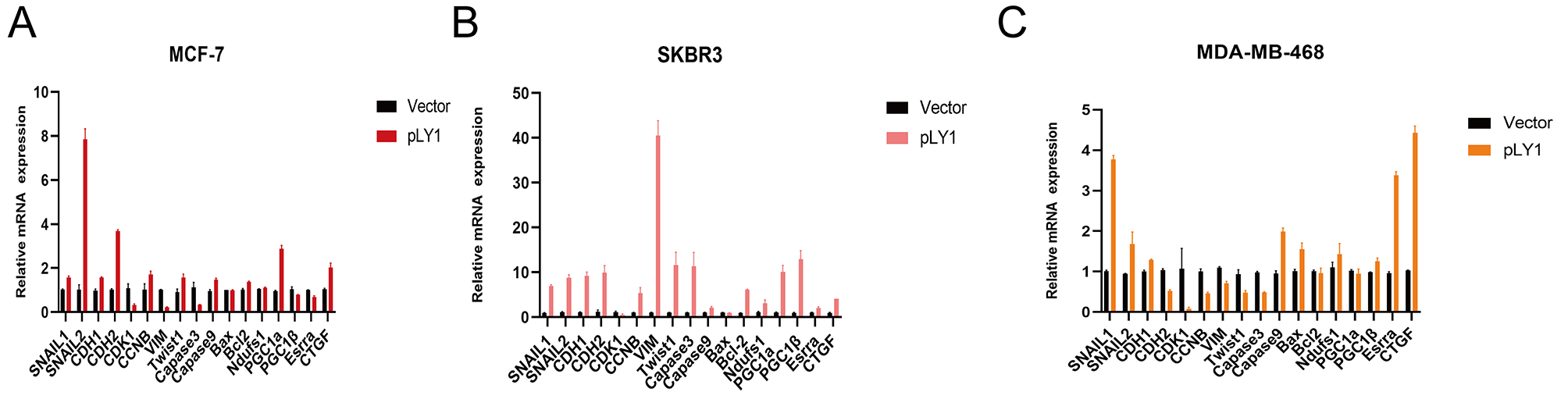

Supplement: Supplementary file 4 — Supplementary Material 4: Supplementary Fig. 4. mRNA expression of related genes in these cells after overexpression of SDHA in MCF-7 (A), SKBR3 (B) and MDA-MB-468 (C) cells. [file 12935_2025_3746_MOESM4_ESM.tiff]
